# Some Curious Legal Nuisances

**Published:** 1892-02

**Authors:** 


### SOME CURIOUS LEGAL NUISANCES.

The following are some of the alleged nuisances for which injunctions and damages have been asked from sympathetic courts and juries:

In a recent English case an enthusiastic amateur played daily eight hours on a violoncello, and on Sundays a little longer some times. To add to the misery caused, the player lived in a flat. The judge before whom the case came decided that three hours was long enough for any human being to play on the violoncello, and the injunction issued.

In a case before the Court of Common Pleas, sometime ago, in New York, a person was brought up for trundling in a carriage overhead his teething baby, both by night and day. The judge, who must have been a married man, held that the noise was not unreasonable, and refused to interfere.

In another English case, a chime of bells at Deptford was not allowed to ring because the noise was offensive to a majority of the property owners in the vicinity.

The newspapers recently mentioned the sad case of a discharged chorister who took a horrible revenge on the congregation by sitting in a pew and purposely singing out of tune. Whether he was indicted or not for disturbing public worship does not appear.

There are few annoying sounds which have not, sooner or later, been alleged as nuisances; but the courts hold that many of them must be endured.—*Medical Record.*

